# The three-dimensional structure of *Clostridium absonum* 7α-hydroxysteroid dehydrogenase: new insights into the conserved arginines for NADP(H) recognition

**DOI:** 10.1038/srep22885

**Published:** 2016-03-10

**Authors:** Deshuai Lou, Bochu Wang, Jun Tan, Liancai Zhu, Xiaoxi Cen, Qingzhi Ji, Yue Wang

**Affiliations:** 1 Key Laboratory of Biorheological Science and Technology (Chongqing University), Ministry of Education, College of Bioengineering, Chongqing University, Chongqing 400030, China; 2 Postdoctoral research station of biology, Chongqing University, Chongqing 400030, China; 3 School of Biological & Chemical engineering, Chongqing University of Education, Chongqing 400067, China

## Abstract

7α-hydroxysteroid dehydrogenase (7α-HSDH) can catalyse the oxidation of C7 α-OH of the steroid nucleus in the bile acid metabolism. In the paper we determined the crystal structure of 7α-HSDH from *Clostridium absonum* (*CA* 7α-HSDH) complexed with taurochenodeoxycholic acid (TCDCA) and NADP^+^ by X-ray diffraction, which, as a tetramer, possesses the typical *α*/*β* folding pattern. The four subunits of an asymmetric unit lie in the fact that there are the stable hydrophobic interactions between *Q*-axis-related subunits. Significantly, we captured an active state of the NADP^+^, confirming that nicotinamide moiety of NADP^+^ act as electron carrier in the dehydrogenation. On the basis of crystal structure analysis, site-directed mutagenesis and MD simulation, furthermore, we find that the guanidinium of Arg38 can form the stable cation-π interaction with the adenine ring of NADP^+^, and the cation-π interaction and hydrogen bonds between Arg38 and NADP^+^ have a significant anchor effect on the cofactor binding to *CA* 7α-HSDH.

In mammals, the primary bile acids, including cholic acid (CA) and chenodeoxycholic acid (CDCA), are synthesised from cholesterol in hepatocyte, and then intestinal bacteria convert primary bile acids to secondary bile acids such as deoxycholic acid (DA), lithocholic acid (LCA) and ursodesoxycholic acid (UDCA). Bile acids/salts and their conjugations with glycine or taurine^1^ achieve an efficient preservation through enterohepatic circulation in the body. In the bile acid metabolism, microbial transformation involves a series of complex processes including epimerization, oxidation, reduction, hydroxylation and dehydroxylation of the steroid nucleus which involves in a variety of microorganisms^2^.

7α-hydroxysteroid dehydrogenases (7α-HSDH) is responsible for reversibly catalysing the oxidation of C7 α-oriented hydroxyl of the steroid nucleus in the bile acid metabolism and has been detected in numerous genera of bacteria^3^,^4^,^5^,^6^,^7^ and in mammal^8^,^9^. To date, three-dimensional structures of two 7α-HSDHs from *Escherichia coli*^10^ and *Brucella Melitensis* have been determined, and the characteristics of folding pattern in both the two 7α-HSDHs indicate that they belong to the short-chain dehydrogenases/reductases (SDRs), whose members are dimeric or tetrameric and generally share low sequence identity possessing a single-domain globular Rossmann-related fold^11^. Based on the cofactor and active site sequence motifs SDRs can be divided to five subfamilies: “classical”, “extended”, “intermediate”, “divergent” and “complex”^12^. All of the 7α-HSDHs reported previously belong to the “classical” subfamily with the cofactor binding site sequence motif ‘TA/G/SxxxGIG’ and the active site sequence motif ‘YxxxK’ (x, any amino acid residue)^12^. Three decades ago, scientists began to try to utilise 7α-HSDH to produce chemical substance with medicinal values^13^,^14^,^15^. Enzyme kinetics data showed 7α-HSDH had a broad substrate specificity. For example, a great variety of chemicals can be identified by 7α-HSDH as substrate such as bile acids and their glycine/taurine conjugate^4^,^6^, benzaldehyde analogues^16^ and aromatic α-ketoesters^17^. Besides, 7α-HSDH present excellent thermal stability that the activity of *Clostridium absonum* 7α-HSDH (*CA* 7α-HSDH) has no significant change after incubation at 25 °C for 108 h^18^; *Bacteroides fragilis* 7α-HSDH (*BF* 7α-HSDH) retains 90% of activity after heat treatment for 1 h at 65 °C^6^. 7α-HSDHs may possess great potential in synthetic application.

All of the dehydrogenases require coenzyme such as NAD(H) or NADP(H) which acts as electron carriers for catalysing the redox reaction, and NADP(H) is much more efficient than NAD(H) in bile acids synthesis process. Therefore, the cofactor specific dehydrogenases attracted a lot of attention. The first crystal structure of NADP(H)-dependent SDR member (mouse lung carbonyl reductase, PDB code: 1CYD) was determined in 1996 and this study^19^ drew a conclusion the key determinant for cofactor specificity were the electrostatic environment surrounding the 2′-phosphate or hydroxyl of the adenosine ribose moiety of NADP(H) or NAD(H). The residues Lys17 and Arg39 are the key residues for mouse lung carbonyl reductase since the two residues could strongly interact with the 2′-phosphate of adenine ribose of NADPH through electrostatic attraction^19^, while the residue Thr38 occupies the spatial position that is similar to a residue Asp of the NAD(H)-dependent SDRs interacting with the hydroxyl groups of the adenine ribose^20^. The T38D mutant successfully switched the cofactor specificity of mouse lung carbonyl reductase confirming the vital importance of the negatively-charged residue at the end of the second *β*-strand for NAD(H)-utilising^20^. Based on the structural information, through primary and tertiary structure comparison, the previous studies presented the basic sequence characteristics of cofactor specificity for SDRs^21^,^22^. Generally, a negative charged (Asp or Glu) residue at the end of the second *β* strand indicates that the enzyme is NAD(H)-preferred and this residue forms hydrogen bonds to the 2′- and 3′-hydroxyl group of the adenine ribose moiety^23^,^24^. NADP(H)-preferred enzymes possess two key basic residues (Arg or Lys). One of them is located at the position just behind the second *β* strand, and the other is positioned just before the sequence ‘GIG’ of the cofactor binding sequence motif mentioned above^19^,^24^. Nevertheless, site-directed mutagenesis performed for altering the cofactor specificity usually cause reduced enzymatic activity^25^ indicating that the determinant of cofactor recognition of SDRs may refer to a space structure involving more than one residue. In biological system, redox reaction prefer to utilising NADP(H). However both the two 7α-HSDHs whose three-dimensional structures were determined up to now utilising NAD(H). The three-dimensional structure the 7α-HSDH utilising NADP(H) has not been determined and their mechanism for cofactor specificity is still not clear.

To unravel the NADP(H) recognition in 7α-HSDH, we determined the crystal structure of *CA* 7α-HSDH complexed with NADP^+^ and taurochenodeoxycholic acid (TCDCA) at resolution 2.0 Å. Site-directed mutageneses were performed to investigate the function of the residues involved in cofactor specificity. Furthermore, molecular dynamics (MD) simulation was carried out to study the interactions between 7α-HSDH and NADP(H)/TCDCA.

## Results and Discussion

### Enzyme purification, crystallisation, diffraction and subunit association

The quality of purified enzyme was verified by sodium dodecyl sulfate polyacrylamide gel electrophoresis (SDS-PAGE) (see Supplementary Fig. S1) and mass spectrometry (MS) (see Supplementary Fig. S2). Size exclusion chromatography (SEC) was applied to determine the molecular weight distribution and the results (see Supplementary Fig. S3) indicated in the *CA* 7α-HSDH mainly existed as dimers. However, seemingly a small proportion (arrows) possesses the association pattern of tetramer (see Supplementary Fig. S3B-1). To verify the association of the subunits of *CA* 7α-HSDH, the dimers from the first SEC were collected and analysed again by SEC (see Supplementary Fig. S3C-1). The tetramers from the two times were determined by MS (see Supplementary Fig. S3B-2 and Fig. S3C-2) and all the molecular weight distributions were corresponding to that of monomer of *CA* 7α-HSDH. It is proposed that a dynamic equilibrium between dimers and tetramers existed in the aqueous solution. The similar oligomerization state was found in the 6-phosphogluconate dehydrogenase from *Trypanosoma brucei*^26^. The crystal of *CA* 7α-HSDH is showed in the Fig. S4. The X-ray diffraction of the crystal is presented in Fig. S5.

### Structure quality assessment

The three-dimensional structure of *CA* 7α-HSDH complexed with NADP^+^ and TCDCA belonged to the *P*2_1_2_1_2_1_ space group and was determined at resolution of 2.0 Å by the molecular displacement method and refined to crystallographic *R*-factor of 0.182 (*R*_free_ = 0.222). For the current model, the backbone dihedral angles of 97.7% of the residues are in the most favored region of the Ramachandran plot, and all the non-glycine residues were in the allowed region. More details of data collection and refinement can be found in Supplementary Table S1 and Table S2, respectively.

### Overall structure

**Figure 1:**
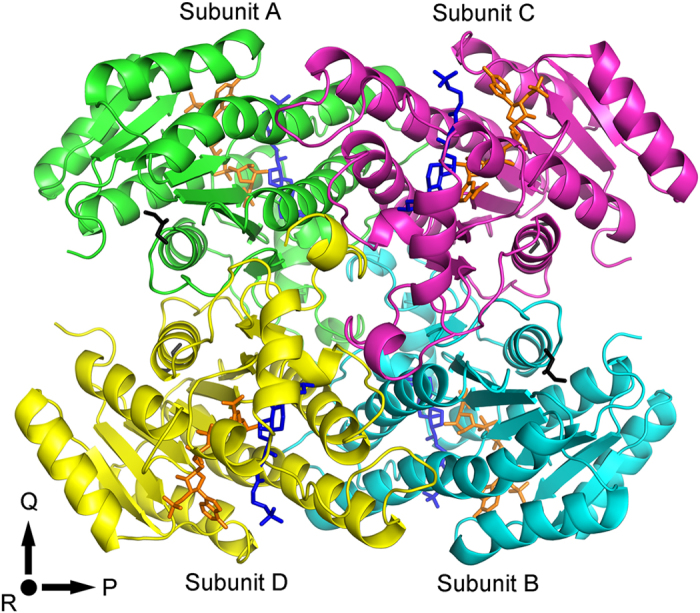
The structure of an asymmetric unit of *CA* 7α-HSDH. TCDCA: blue stick; NADP^+^: orange stick; Glycerol: black stick.

An asymmetric unit of *CA* 7α-HSDH crystal structure includes four subunits (Fig. 1). There is no major difference for the four subunits through pair-wise comparison (Main-Chain RMSD values ranged from 0.27 to 0.34 Å). Additionally, the glycerols in the crystal are from the cryo-protection solution.

To date, four three-dimensional structures of 7α-HSDHs have been deposited in PDB database. They are *Brucella Melitensis* 7α-HSDH with no ligand (PDB code: 3GAF) at resolution 2.20 Å and *Escherichia coli* 7α-HSDH (*EC* 7α-HSDH; PDB code: 1FMC, 1AHI and 1AHH). Structure of 1AHH contains ligand NAD^+^ at resolution 2.30 Å, while both 1FMC and 1AHI have the ligands NADH and 7-oxo-GCDCA at resolution 1.80 Å and 2.30 Å, respectively. In spite of the low sequence identity (only 15–30%), SDRs generally display the well-conserved Rossmann fold. As one of the SDRs, with the similar structure to the two 7α-HSDHs above, *CA* 7α-HSDH possesses the typical Rossmann-fold that each monomer consists of seven parallel *β*-strands as a core (*β*-sheet core) and *α*-helices located between *β*-strands at the surface of the globular molecules. The three-dimensional structures and folding topologies of *CA* 7α-HSDH and *EC* 7α-HSDH are shown as Supplementary Figs S6 and S7, respectively. Although the two conformations are very similar, there are some structural differences in detail. *CA* 7α-HSDH has longer C-terminal segment including an extra α-helix (αI) which involves Gln255-Tyr256-Ser257-Glu258-Tyr259. Differing from that of *EC* 7α-HSDH, such C-terminal structure of *CA* 7α-HSDH does not directly surround the substrate-binding pocket but stretch outward. We have previously proved that only 19.1% of catalytic efficiency (*k*_cat_/*K*_m_) remained after truncation of 2 amino acids at C-terminal end of *CA* 7α-HSDH^18^. Therefore, the C-terminal segment has a great influence on the catalytic activity of *CA* 7α-HSDH.

**Figure 2:**
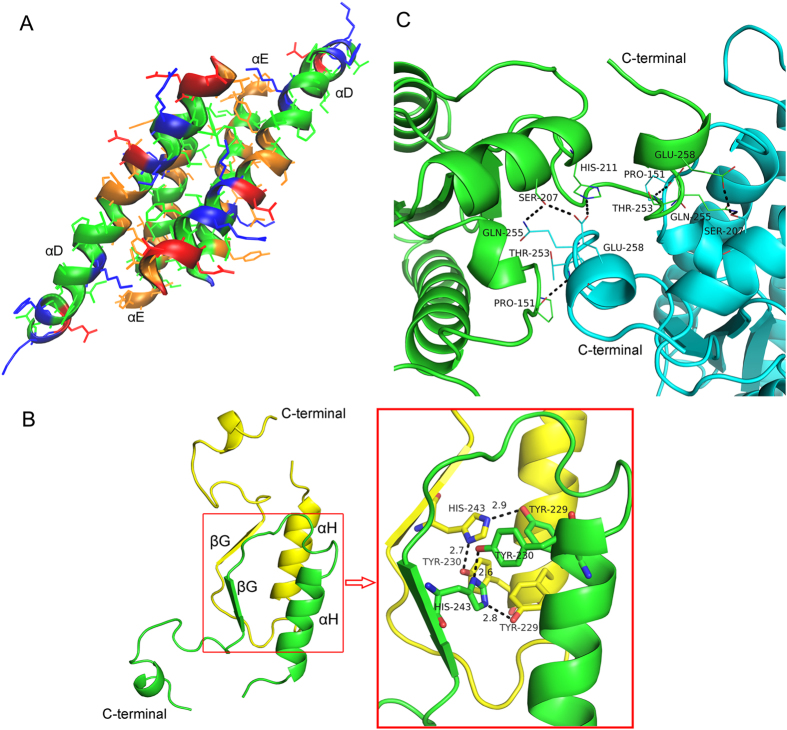
The Overall structure and the interactions in the subunits. (**A**) Helix D and E mediate interactions in *Q*-axis-related subunits (subunit A and C, B and D). blue: basic residues; red: acidic residues; orange: polar residues; green: nonpolar residues. (**B**) The C segment mediates interactions in *P*-axis-related subunits. Green: subunit A; yellow: subunit D. (**C**) The polar interactions between subunits in the diagonal position of an asymmetric unit structure of *CA* 7α-HSDH.

The four subunits in an asymmetric unit of *CA* 7α-HSDH are shown in Fig. 1. Following the naming proposed by Ghosh *et al.*^27^ the three mutually perpendicular directions are named *P*, *Q* and *R* axes, respectively. Along *Q*-axis, the interactions between subunit A and C (B and D) are mediated by four α-helices, αD (residues 107–135) and αE (residues 156–176), which form αD-αD and αE-αE interfaces. As shown in Fig. 2A, the side chains of hydrophobic residues Phe110, Phe111, Phe114, Val118, Val121, Ala125, Ile129, Ile156, Ala157, Ile165, Leu168 and Ile172 are embed in the interspaces among the four helices, resulting in the stable hydrophobic interactions between subunit A and C (B and D). Similar *Q*-axis related interactions exist in the crystal structure of *EC* 7α-HSDH^10^. The interactions between *P*-axis related subunits (subunits A and D, or subunits B and C) mainly result from *α*H (residues 220–231) and *β*G (residues 242–245). Figure 2B shows the interaction pattern between subunit A (green) and subunit D (yellow). Phenolic hydroxyls of the residues Tyr230 A and Tyr229 D form hydrogen bonds with the two nitrogen atoms of imidazole ring in the residue His243 A, while phenolic hydroxyls of the residues Tyr230 D and Tyr229 A form hydrogen bonds with the two nitrogen atoms of imidazole ring of His243 D, and vice versa. This particular interaction pattern may play an important role in the subunit association along *P*-axis. In the aqueous solution, salvation effect could weaken the interaction between *P*-axis related subunits, resulting in that a multitude of tetramers dissociated into the dimmers. This well explains the dynamic equilibrium between dimers and tetramers of *CA* 7α-HSDH in the aqueous solution mentioned above.

In an asymmetric unit, the polar interactions between the two subunits in the diagonal position (subunits A and B, subunits C and D) involves the C-terminal as shown in Fig. 2C. The residues Glu258, Gln255 and Thr253 in one subunit form hydrogen bonds with the residues His211/Ser207, Ser207, Pro151 in the other subunit, respectively. The similar interactions mediated by C-terminal could be found in the asymmetric unit (four subunits) of 3α, 20β-HSDH from *Streptomyces exfoliatus* (PDB code: 2HSD)^28^.

In addition, we found many salt bridges exist in the surface of the monomer such as Asp49-Arg20, Asp85-Arg83, Asp152-Arg155, Asp202-Arg205. The thermostability of *CA* 7α-HSDH at 25 °C^18^ may be attributed to these salt bridges.

**Figure 3:**
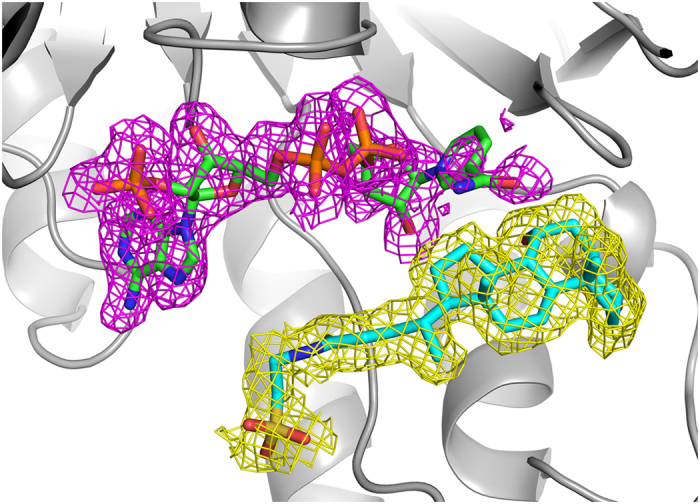
Electron density map (|*F*_o_|−|*F*_c_| omit map) contoured at 1.0σ. TCDCA is presented with yellow mesh and NADP^+^ with red, respectively.

For the molecular NADP^+^, electron density of the nicotinamide moiety of NADP^+^ was rather weak (Fig. 3) suggesting this part was in an active state, which confirmed that nicotinamide moiety of NADP^+^ act as electron carrier in the dehydrogenation reaction.

### The active site of *CA* 7α-HSDH

**Figure 4:**
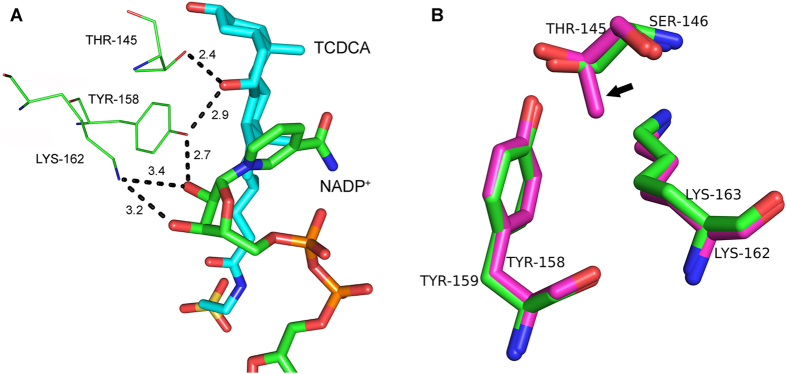
The active site. (**A**) The polar interactions between active sites and NADP^+^/TCDCA in *CA* 7α-HSDH. (**B**) The superposed architecture of active sites of *CA* 7α-HSDH (red carbon atoms) and *EC* 7α-HSDH (green carbon atoms). An arrow indicates the Thr145 methyl of *CA* 7α-HSDH.

The well-conserved active sites with the sequence motif, YxxxK, exist in the three subfamilies (classical, extended and intermediate) of SDRs, and the tyrosine is the most conserved residue which exists in all members of SDRs^12^,^29^. Generally, a serine located before the tyrosine also play an important role in the catalytic process forming the Ser-Tyr-Lys triad. The active site in *EC* 7α-HSDH, consists of Ser146, Tyr159 and Lys163, and the proposed catalysis mechanism suggested the residue Tyr159 might act as a catalytic base through deprotonation of phenolic group and the hydrogen bond with C7 hydroxyl of substrate while the Ser and Lys stabilise the substrate and cofactor respectively through hydrogen bonds^10^. Furthermore, the site-directed mutageneses of these three residues dramatically decrease the enzyme activity especially of the mutant of Tyr159^30^. Sequence alignment^31^ suggested the Ser-Tyr-Lys triad is well conserved in five 7α-HSDHs known to date but not in the *CA* 7α-HSDH. However, *CA* 7α-HSDH possesses the active site, Thr145-Tyr158-Lys162, rather than Ser-Tyr-Lys triad. Here, the evolutionarily conserved serine was substituted by threonine. Interestingly, such mutation does not exist in the *Clostridium absonum* 7β-HSDH (*CA* 7β-HSDH). Figure 4A showed the interactions between active sites and NADP^+^/TCDCA and the distances (Å) between relative atoms in *CA* 7α-HSDH. The residue Thr145 forms hydrogen bond with C7-OH of TCDCA while the residue Lys162 forms hydrogen bonds with 2′- and 3′-hydroxyl group of the nicotinamide ribose moiety in NADP^+^. The phenolic hydroxyl of Tyr158 form hydrogen bonds with C7-OH of TCDCA and 2′-hydroxyl group of the nicotinamide ribose of NADP^+^. To observe difference of active-site spatial arrangement between *CA* 7α-HSDH and *EC* 7α-HSDH, we superposed the two catalytic triads (Fig. 4B). Although threonine has an additional methyl comparing with serine, no prominent difference of spatial placement exists for hydroxyl groups of threonine and serine in the active site. Thus, the mutation of serine to threonine during evolution does not cause the complete activity loss of *CA* 7α-HSDH.

### Cofactor-binding site

**Figure 5:**
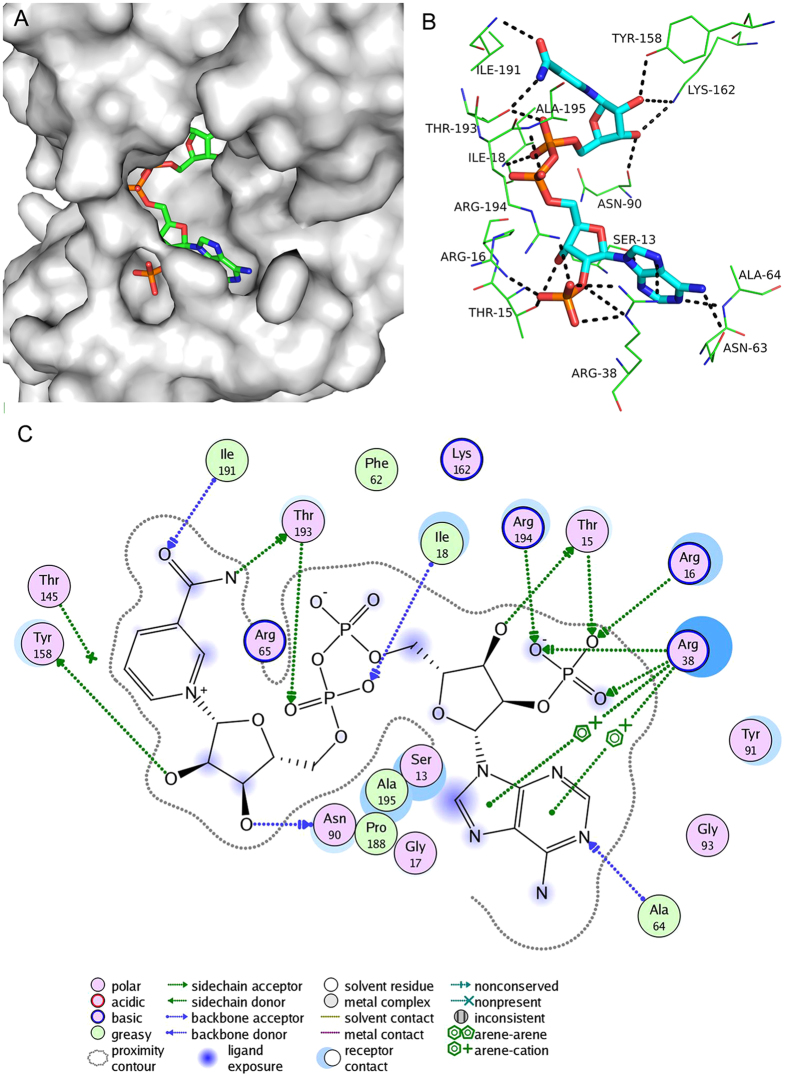
The cofactor-binding site. (**A**) The location and orientation of NADP^+^. (**B**) The key interactions and H-bond patterns between NADP^+^ and *CA* 7α-HSDH. (**C**) The related polar interactions.

On account of the conserved hydrogen bonding of cofactor onto protein main chain of SDRs^32^, the conformation and orientation of NADP^+^ in *CA* 7α-HSDH are similar to that of other NADP(H)-dependent SDRs such as human estrogenic 17β-HDSH^32^ (PDB code: 1QYV), trihydroxynaphthalene reductase^33^ (PDB code: 1DOH) and tropinone reductase^34^ (PDB code: 2AE2). Figure 5 showed the multiple interactions between *CA* 7α-HSDH and its cofactor NADP^+^ mainly involving Ser13, Thr15, Arg16, Ile18, Arg38, Asn63, Asn90, Tyr158, Lys162, Ile191, Thr193, Arg194, Ala195. Thr15, Arg16, Arg38 and Arg194 formed a positive charge-rich environment surrounding the 2′-phosphate of adenine ribose and these residues determined the cofactor specificity in *CA* 7α-HSDH. According to the theory mentioned above, Arg16 and Arg38 are the determinant residues for cofactor specificity. In previous study, we performed the mutation analysis to verified the function of Arg38 of *CA* 7α-HSDH in the cofactor recognition and found that the R38D mutant did not display enzyme activity with either NADP^+^ or NAD^+ ^18. Here, to investigate the function of the residues Arg16, Ala37 and Arg38, nine mutants of *CA* 7α-HSDH were prepared as follows: A37D, R16G, R16G/A37D, R16G/R38D, A37D/R38I, A37D/R38Y, A37D/R38V, R16G/A37D/R38I and R16G/A37D/R38V. Interestingly, the results of enzyme assay indicate that only the mutant R16G has enzyme activity with NADP^+^ and no activity with NAD^+^. No activity of the other eight mutants was detected with either NADP^+^ or NAD^+^. Such results show the alteration of cofactor specificity in *CA* 7α-HSDH is more complex and implementation of the residue with negative charges (aspartic acid) at position 37 or 38 could not form proper space orientation for NAD(H) recognition. From a structural point of view, the three arginines in the position 16, 38 and 194 form a positive environment surrounded the 2′-phosphate of adenine ribose of NADP^+^ in *CA* 7α-HSDH, but not more than two arginines were at the corresponding position in the other 10 SDRs listed as Supplementary Table S3.

Furthermore, the amino groups in Arg16, Arg38 and Arg194 form strong hydrogen bonds with the oxygen atoms in the 2′-phosphate of adenine ribose of NADP^+^. Especially, the nitrogen atom in guanidinium of Arg38 is close (~4 Å) to the adenine ring of NADP^+^ forming the cation-π stacking. Cation-π interactions are ubiquitous in biological systems, which play central roles in molecular recognition, stabilisation of protein structures and nucleic acid structures, and their biological functions. Cheng *et al.*^35^ studied the cation-π interactions between NH_4_^+^ and benzene by the density function calculation, finding that the energy difference between the lowest unoccupied molecular orbital (LUMO) of NH_4_^+^ and the highest occupied molecular orbital (HOMO) of benzene is more than that between LUMO of benzene and HOMO of NH_4_^+^. So the electrons preferentially move from the benzene to NH_4_^+^ combined with the analysis of net charge of atom. We speculate that the cation-π interactions might lead to the electrons of the adenine ring of NADP^+^ moving to the NH_2_^+^ of Arg38.

To investigate the effects of this electrostatic environment on NADP^+^ binding, the mutants, R16G and R194G, were prepared and the enzyme activities were determined. Table 1 presents the kinetics constants of WT, R16G and R194G toward cofactor NADP^+^. The *k*_cat_ and *K*_m_ of R16G mutant increase by more than four times and five times compared with those of WT, respectively, while the catalytic efficiency (*k*_cat_/*K*_m_) of R16G mutant decrease by 17.26%. The increase in *K*_m_ indicates that affinity of R16G mutant toward NADP^+^ become weak, while the cofactor NADP(H) dissociated more easily from the binding site resulting in the increase in *k*_cat_. Moreover, similar change of *K*_m_ value also occurred in the R194G mutants. Hence, the formation of the electrostatic environment which mainly involves the 3 arginines (Arg16, Arg38 and Arg194) mediates the cofactor specificity and recognition in right orientation.

### The key role of arginine residue

To determine the role of the arginine residues Arg16 and Arg38, molecular dynamics (MD) simulations were performed to investigate the dynamics of ternary complexes of wild type, mutant R16G and R38D. The conformational stability was verified by root mean square deviation (RMSD) of the main chain of protein compared with the initial structure of protein (see Supplementary Fig. S8). For ternary complexes WT-TCDCA-NADP^+^ (WT-ternary), R16G-TCDCA-NADP^+^ (R16G-ternary), R38D-TCDCA-NADP^+^ (R38D-ternary), during 5ns MD simulations the RMSD of the backbone achieved stability quickly and kept fluctuation range from 1.0 to 1.5Å. Based on MD simulations, Molecular Mechanics Poisson Boltzmann Surface Area (MM/PBSA) calculations were preformed to evaluate the binding energies between enzymes and ligands in the three systems mentioned above. The plasticity of WT-ternary, R16G-ternary and R38D-ternary was evaluated through root mean square fluctuations (RMSF) (see Supplementary Fig. S9), and the results indicated that the catalytic site (T145-Y158-K162) was significantly rigid in all the systems. G54 and T103 had higher plasticity in WT-ternary and R16G-ternary respectively, and the same trend of R65 and I153 in the mutate R38D-ternary was observed. Nevertheless, the backbone plasticity was not remarkably influenced by the two mutations R16G and R38D.

**Figure 6:**
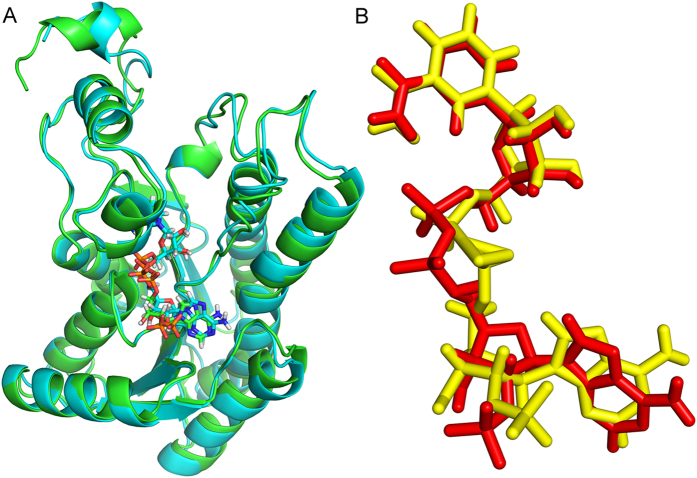
Structural alignments of WT-ternary and R16G-ternary. (**A**) Overall structural alignment (WT: cyan; R16G: green). (**B**) Cofactor alignment (NADP^+^ of WT: red; NADP^+^ of R16G: yellow).

**Figure 7:**
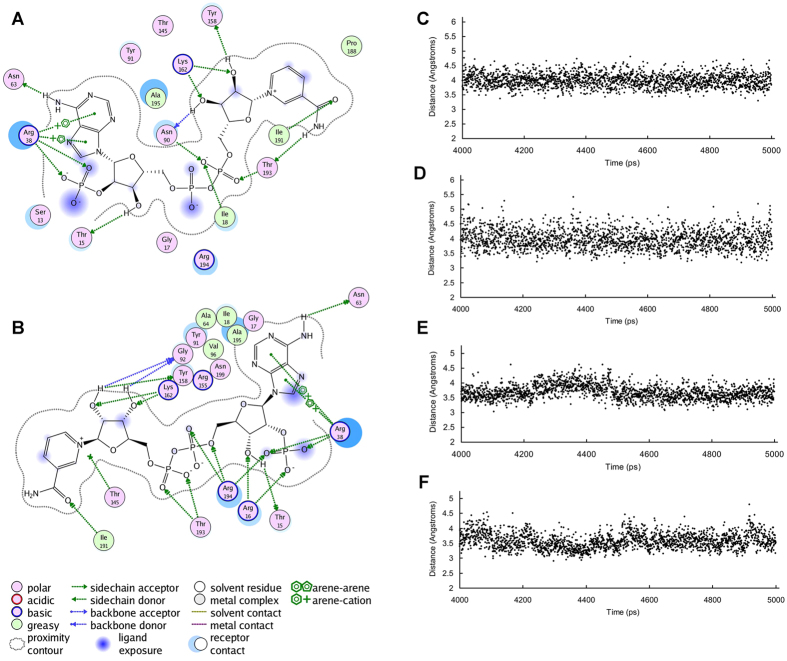
The interactions between NADP^+^ and *CA* 7α-HSDH. Residues involving in interactions between NADP^+^ and WT-ternary (**A**) R16G-ternary (**B**). The distances between NE (**C**) NH_2_ (**D**) of guanidinium of Arg38 of WT- ternary and adenine ring of NADP^+^ in the cation-π interactions. The distances between NADP^+^ and Arg38 of mutant R16G- ternary and NE (**E**), NH_2_ (**F**) of Arg 38 and adenine ring of NADP^+^ in cation-π interactions.

Furthermore, the average structures of last 1ns of the WT-ternary and R16G-ternary systems were calculated and aligned. Figure 6 presented the overall structural superposition suggesting no obvious conformational difference existed between the enzymic average structures of WT and that of the mutant except for the C-terminal. We find that the hydrogen bonds between Arg16, Arg38 and Arg194 and the oxygen atoms in the 2′-phosphate of adenine ribose of NADP^+^ in the WT-ternary structure remain stable and the donor-acceptor distances of these H-bonds remain mostly between 2.7 Å and 3.1 Å during 5 ns dynamics (see Supplementary Fig. S10). Furthermore, we find that the distances between =NH_2_^+^ of guanidinium of Arg38 of WT-ternary and adenine ring of NADP^+^ mostly fluctuate between 3.5 Å and 4.5 Å, indicating that the cation-π interactions between Arg38 and NADP^+^ are also stable (Fig. 7).

After the mutation of arginine at postion16 to glycine, both Gly16 and Arg194 do not form the stable hydrogen bond with NADP^+^, and the pyrophosphate bridge slightly rotation, which is attributed to the change of the surroundings of 2′-phosphate of adenine ribose of NADP^+^ in the R16G mutant. Furthermore, we find that the hydrogen bond between Arg38 and 2′-phosphate of adenine ribose of NADP^+^ still exsits, but the H-bond is very weak because the donor-acceptor distances of the H-bond remain mostly between 3.2 Å and 3.8 Å during 5 ns dynamics (see Supplementary Fig. S11). The distances between =NH_2_^+^ of guanidinium of Arg38 of R16G-ternary and adenine ring of NADP^+^ mostly fluctuate between 3.2 Å and 4.0 Å (Fig. 7). Although the stability of the H-bond between Arg38 and NADP^+^ in the R16G-ternary decreased, the cation-π interactions enhanced, compared with those of WT-ternary. Fortunately, the nicotinamide moiety keeps in the original position (Fig. 6B), so the R16G mutant still has strong enzyme activity. The MM/PBSA results indicated that the mutation does not influence the binding of TCDCA to enzyme-cofactor complex, while the binding energy of NADP^+^ to enzyme-substrate complex significantly increased from −99.84 to −72.99 kcal·mol^−1^ after the mutation of arginine at postion16 to glycine (see Supplementary Table S4).

After the mutation of arginine at postion 38 to aspartic acid, the hydrogen bonds between Arg16 and NADP^+^ remain stable, which is similar to the WT-ternary. In the mutant R38D without any activity, however, the hydrogen bond and cation-π interactions between the residue at position 38 and NADP^+^ are missing, resulting in the wide fluctuation of the adenine ring of NADP^+^. So the arginine at postion 38 in the WT-ternary and the R16G-ternary can anchor the adenine ring of NADP^+^ in the appropriate position through the cation-π interactions and hydrogen bonds.

As seen in MD simulation results, it is not hard to get the following points of view. i) The correct binding position of nicotinamide moiety which directly involved in the oxidation-reduction reaction of NAD(P)(H) is the key determinant of the activity of R16G, and the correct spatial orientation ensure that the catalytic sites could form the proper interaction with substrate and cofactor. ii) The rotation of the pyrophosphate moiety of NADP(H) indicates that the flexibility of the pyrophosphate bridge may allow the shaking of nicotinamide moiety for electron transfer. However, molecular modification of *CA* 7α-HSDH (such as site-directed mutageneses of A37D and R38D) may remarkably change the NADP(H) binding pattern that nicotinamide moiety deviates the right position for interactions with substrate-enzyme. iii) Although the side-chain removal of Arg16 changed the electrostatic surroundings constructed by Arg16–Arg38–Arg194, the hydrogen bonds between 2′-phosphate of adenine ribose and Arg38 remained (Fig. 7) in the both WT and R16G systems. Once Arg38 was removed, the mutant R38D would lose enzyme activity. We conclude that the Arg38 is the key determinant of NADP(H) recognition in *CA* 7α-HSDH. Such MD simulation result corresponded to that of mutagenesis experiments mentioned above. iv) The anchor effect of Arg38 though cation-π interactions with adenine moiety may play an important role in the cofactor binding to *CA* 7α-HSDH. Similar interactions could be observed in some of SDRs, such as *Sus scrofa* carbonyl reductase/20 beta-hydroxysteroid dehydrogenase and *Mus musculus* Sepiapterin reductase (see Supplementary Fig. S12).

## Conclusion

We showed the crystal structure of *CA* 7α-HSDH including four subunits. *CA* 7α-HSDH, as a member of SDRs, possesses the typical *α*/*β* folding pattern and the very similar topological structure to *EC* 7α-HSDH. Nevertheless, differing from *EC* 7α-HSDH, *CA* 7α-HSDH is NADP(H)-dependent and has a more complex C-terminal structure. The tetramer of *CA* 7α-HSDH lies in the fact that there are the stable hydrophobic interactions between subunit A and C (B and D) and the hydrogen bonds between subunit A and D (B and C). Especially, the latter fixes the four subunits of *CA* 7α-HSDH, but in the aqueous solution the hydrogen bonds between subunit A and D (B and C) could be weaken by salvation effect, resulting in the dissociation of the tetramer to the dimer. We further find that the residue Thr145 performs hydrogen abstraction through the hydrogen bond with C7-OH of TCDCA, while the serine executes the same function in the other SDRs. So the active residues related to the dehydrogenation catalysis involve in Thr-Lys-Tyr combination in *CA* 7α-HSDH, differing from the Ser-Lys-Tyr combination in the other SDRs.

Significantly, we captured an active state of the coenzyme NADP^+^ with the rather weak electron density of the nicotinamide moiety, which confirmed that nicotinamide moiety of NADP^+^ act as electron carrier in the dehydrogenation reaction. In the cofactor-binding site, the three arginines at the position 16, 38 and 194 form a positive environment surrounded the 2′-phosphate of adenine ribose of NADP^+^ in *CA* 7α-HSDH. The residues Arg16, Arg38 and Arg194 can form strong hydrogen bonds with the oxygen atoms in the 2′-phosphate of adenine ribose of NADP^+^. Especially, the guanidinium of Arg38 with positive charge can form the cation-π interaction with the adenine ring of NADP^+^. On the basis of crystal structure analysis, site-directed mutagenesis and MD simulation, we find that the cation-π interaction and hydrogen bonds between Arg38 and NADP^+^ have a significant anchor effect on the cofactor binding to *CA* 7α-HSDH. So we strongly believe that the residue Arg38 play an important role in the dehydrogenation reaction mediated by *CA* 7α-HSDH.

## Methods

### Heterologous expression and purification of *CA* 7α-HSDH

*CA* 7α-HSDH gene was cloned into plasmid pGEX-6p-1 and the GST-fused enzyme was heterologously expressed in *E. coli* BL21 (DE3) using the previously reported method^18^. The preliminarily purified enzyme was further purified for the protein crystallisation as followings. Enzyme was loaded onto a buffer (50 mM Tris-HCl pH8.0, 50 mM NaCl, 5% Glycerol, 5 mM DTT) pre-equilibrated monoQ 10/100 GL (GE Healthcare) column and 30 column volumes 0–100% buffer (50 mM Tris-HCl pH8.0, 1 M NaCl, 5% Glycerol, 5 mM DTT) was employed to wash and elute the enzyme. Then, the enzyme was loaded onto the buffer (50 mM Tris-HCl pH8.0, 200 mM NaCl) pre-equilibrated superdex 75 column (GE Healthcare). 1.2 column volumes of buffer C were used to wash and elute column and the purified enzyme was collected. Finally, the samples were verified by SDS-PAGE and MS. High purity enzyme was concentrated to a proper concentration for crystallisation experiments. Molecular weight distribution of *CA* 7α-HSDH in aqueous solution was analysed through size exclusion chromatography (SEC) with standard protein (GE Healthcare) molecular weight 669, 440, 158, 75, 44, 29 and 13.7 kDa.

### Crystallisation

Crystal screening using a crystallisation robot was performed at 293 K by sitting-drop vapour-diffusion method. 200 nL protein solution was mixed with 200 nL reservoir solution and equilibrated against 30 μL reservoir solution. The concentrations of enzyme, NADP^+^ and TCDCA in protein solution are 1.12 mM, 5.6 mM and 5.6 mM, respectively. Commercial crystallisation kits from Hampton Research were used for crystal screening. Initial crystals of complex were obtained in 0.1 M HEPES (pH 7.5) buffer containing 25% PEG-3350.

### Data collection and refinement

The crystal was transferred to the cryo-protection solution (0.1 M HEPES pH 7.5, 25% PEG-3350, 25% Glycerol). Immediately, we mounted the crystal onto the goniometer which was surrounded by nitrogen at 100 K and all of the data collection works by using FR-E^+^ and R-AXIS IV of RIGAKU. The structure of *EC* 7α-HSDH (PDB code: 1FMC) was used as model for solving the *CA* 7α-HSDH structure by molecular replacement techniques. The software iMosflm^36^ used for the data processing and the software Phaser^37^ was employed to phasing the crystal structure. The model building and refinement were through software REFMAC^38^, PHENIX^39^ and Coot^40^. Software PHENIX^39^ and PROCHECK^41^ were used for structure validation and reporting MolProbity clashscores and rotamers. PyMOL were used for picture making. The structure of *CA* 7α-HSDH has been deposited into the PDB (PDB code: 5EPO).

### Molecular dynamic simulations

In the asymmetric unit (4 subunits) of *CA* 7α-HSDH crystal, Initial structure of subunit A, NADP^+^ and TCDCA were extracted for molecular dynamic simulation. Initial coordinates of Arg16Gly mutant was prepared by virtual mutation and structurally minimised using SYBYL program (TRIPOS Inc., St. Louis, MO). Wild-type ternary complex (WT-TCDCA-NADP^+^) or mutant ternary (R16G-TCDCA-NADP^+^) was immersed in TIP3 water box. Na^+^ ions were added to neutralise negative charges of the system, then, topology and coordinate files were created by LEaP module. Three rounds of minimisation were carried out: (1) 4,000-step minimisation of solvent and ions; (2) 5,000-step minimisation of solution and side chains; (3) 10,000-step minimisation of whole system. Combination of steepest descent and conjugate gradient method was applied in these minimisations. Temperature of system increased from 0 K to 300 K within 50 ps MD simulation. Then, another 50 ps MD simulation in NPT ensemble and 5 ns final MD simulation were performed at constant temperature (300 K). The Amber Molecular Dynamics Package (AMBER12) was used for all the MD simulation^42^.

### Site-directed mutagenesis

Table S5 (see Supplementary) summarised the mutagenic forward primers used for site-directed mutagenesis using overlapping PCR for the mutants: A37D, A37D/R38I, A37D/R38Y and A37D/R38V. On the basis of the mutants above, a series of mutants R16G, R16G/R38D, R16G/A37D, R16G/A37D/R38I and R16G/A37D/R38V were performed using conventional PCR with the forward primer 5′-CGGGATCCATGAAACGCCTGGAAGGCAAAGTGGCAATTGTGACCAGCTCTACTGGTGGCATTGGC-3′ and the reverse primer 5′-CTTTTGCGGCCGCTTAGCGCGGGCAGTATTC-3′. These mutants mentioned above were performed by conventional or overlapping PCR with primeSTAR HS DNA polymerase (TaKaRa, Dalian, China) and all the genes were verified by sequencing (TaKaRa, Dalian, China). The gene of mutant R194G was synthesised and verified at Sangon Biotech (Sangon, Shanghai, China).

### Assay of enzyme activity

Enzyme assay and kinetic analysis of wild-type *CA* 7α-HSDH and the mutants were determined by the previously reported methods^18^,^43^.

## Additional Information

**How to cite this article**: Lou, D. *et al.* The three-dimensional structure of *Clostridium absonum* 7α-hydroxysteroid dehydrogenase: new insights into the conserved arginines for NADP(H) recognition. *Sci. Rep.* **6**, 22885; doi: 10.1038/srep22885 (2016).

## Supplementary Material

Supplementary Information

## Figures and Tables

**Table 1:** Kinetics constants of WT, R16G and R194G toward NADP^+^.

**Enzyme**	***k***_**cat**_**(s**^**−1**^)	***K***_**m**_**(mM)**	***k***_**cat**_**/*****K***_**m**_**(s**^**−1**^ **mM**^**−1**^)
WT	13.76 (0.74)	0.34 (0.01)	40.10
R16G	58.55 (2.32)	1.77 (0.13)	33.18
R194G	28.66 (1.86)	0.72 (0.06)	39.78

WT: wild type; R16G: the mutant of arginine at position 16 to glycine; R194G: the mutant of arginine at position 194 to glycine; The values of standard deviations (SD) are in parentheses.
