# Supplementary material for: The three-dimensional structure of Clostridium absonum 7α-hydroxysteroid dehydrogenase: new insights into the conserved arginines for NADP(H) recognition
Source: Sci Rep. 2016 Mar 10;6:22885. doi: 10.1038/srep22885 (PMC4785404; doi:10.1038/srep22885)
Supplement: Supplementary Information [file srep22885-s1.pdf]

The three-dimensional structure of *Clostridium absonum* 7α-hydroxysteroid dehydrogenase: new insights into the conserved arginines for NADP(H) recognition

Deshuai Lou[1,2], Bochu Wang[1,*], Jun Tan[1,3,*], Liancai Zhu[1], Xiaoxi Cen[1], Qingzhi Ji[1], Yue Wang[1]

[1] Key Laboratory of Biorheological Science and Technology (Chongqing University), Ministry of Education, College of Bioengineering, Chongqing University, Chongqing 400030, China

[2] Postdoctoral research station of biology, Chongqing University, Chongqing 400030, China

[3] School of Biological & Chemical engineering, Chongqing University of Education, Chongqing 400067, China

* Corresponding. wangbc2000@126.com (Bochu Wang) and tanjunmail@126.com (Jun Tan)

Supplementary Information

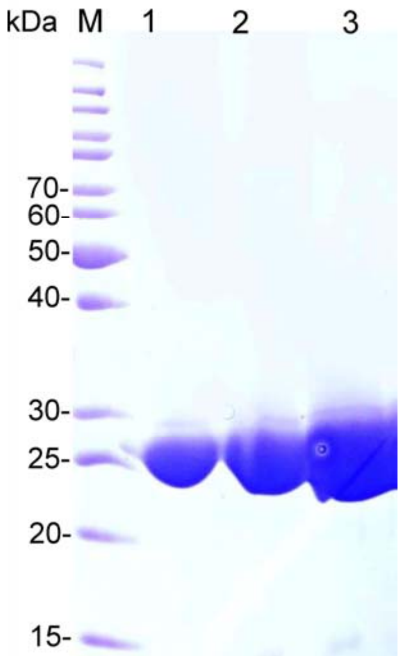

Fig. S1. SDS-PAGE of purified 7α-HSDH from *Clostridium absonum*. M: marker; Lane 1: 5μg; Lane 2: 10μg; Lane 3: 20μg.

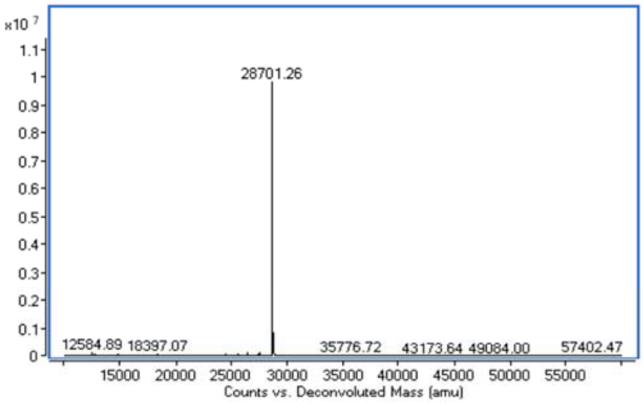

Fig. S2. Mass spectrum verification of 7α-HSDH from *Clostridium absonum*.

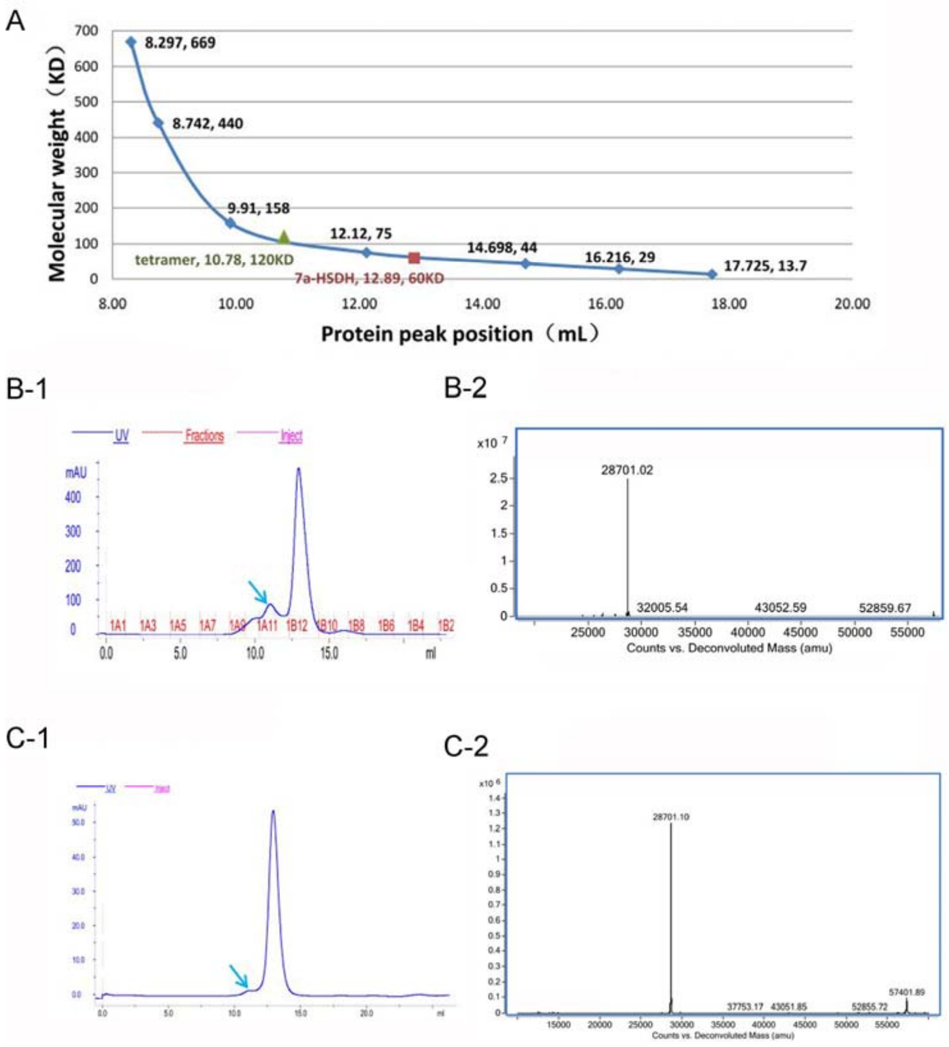

Fig. S3. SEC analysis of 7α-HSDH from *Clostridium absonum* (*A*) The retention times of standard proteins. (*B-1*) Protein peaks of the first SEC. (*B-2*) The first MS analysis. (*C-1*) Protein peaks of the second SEC. (*C-2*) The second MS analysis.

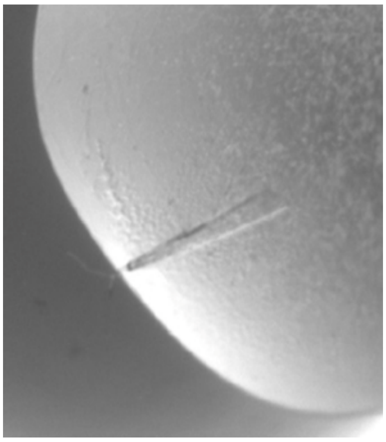

Fig. S4. Crystal image of 7α-HSDH from *Clostridium absonum*.

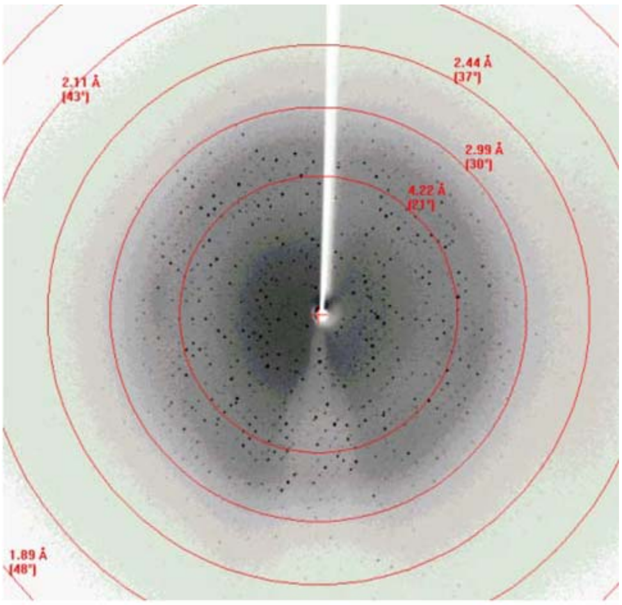

Fig. S5. Diffraction image of the crystal of 7α-HSDH from *Clostridium absonum*.

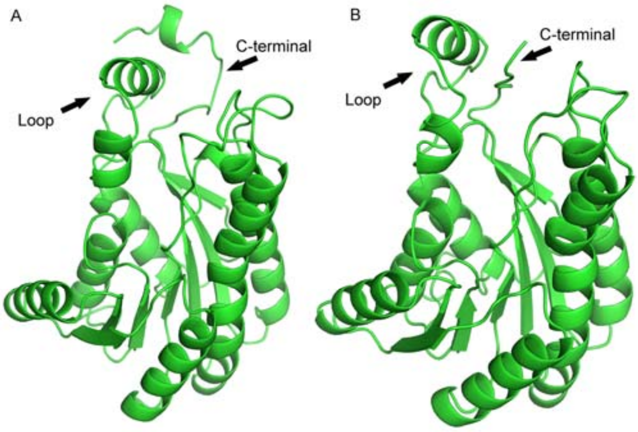

Fig. S6. Comparison of three-dimensional structures. (*A*) *CA* 7α-HSDH. (*B*) EC 7α-HSDH (PDB: 1FMC).

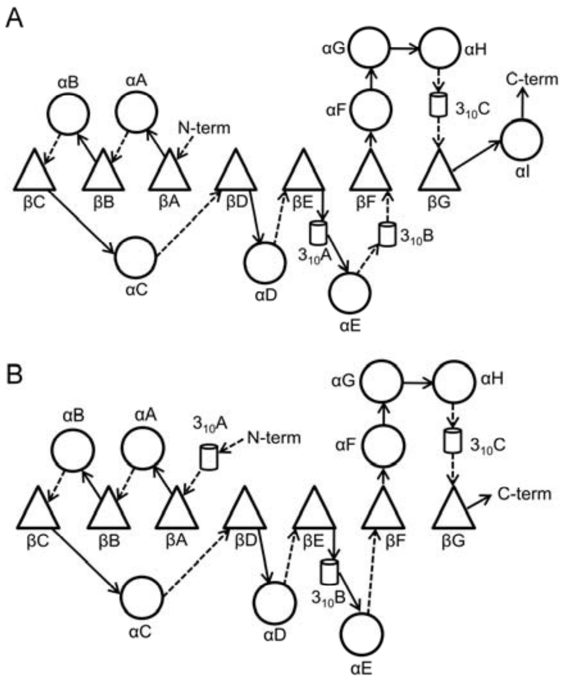

Fig. S7. Comparison of folding topologies. (A) *CA* 7α-HSDH. (B) *EC* 7α-HSDH. Circles, cylinders and triangles stand for α-helix, 3_{10}-helix and β-strands respectively.

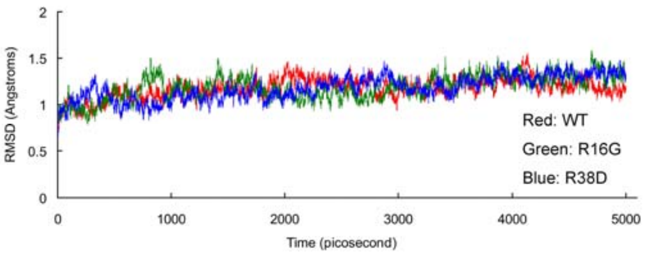

Fig. S8. The RMSDs of backbone atom comparing with the initial structures during 5ns MD simulations.

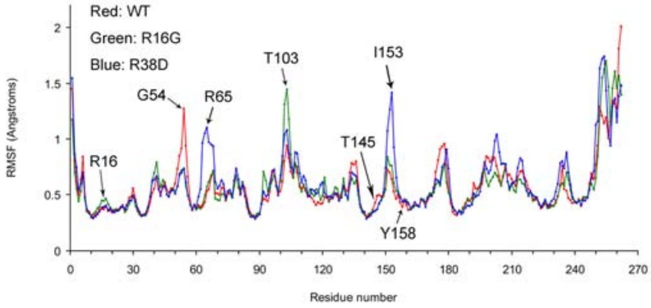

Fig. S9. The backbone RMSF of WT, R16G and R38D.

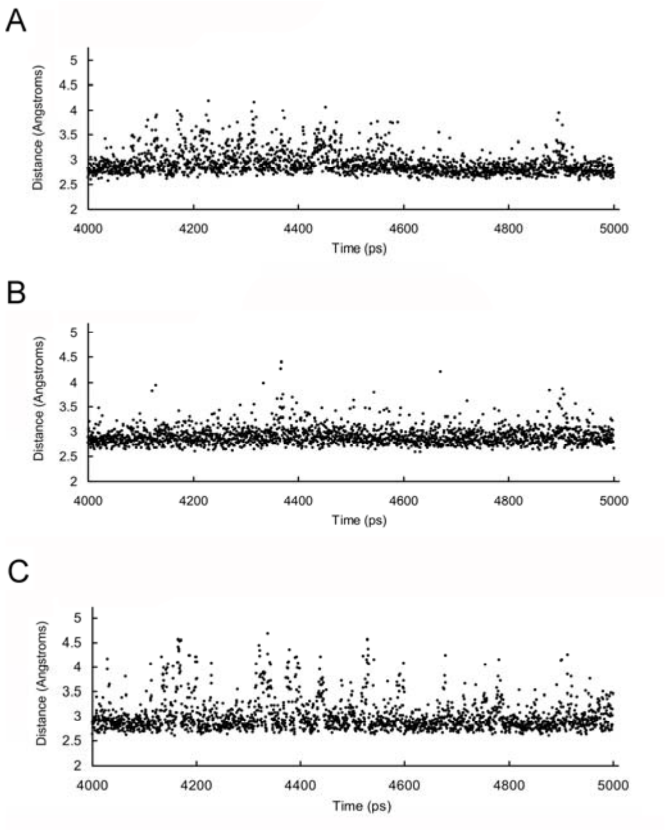

Fig. S10. Donor-acceptor distances of H-bonds between the 2′-phosphate of NADP(H) and the guanidiniums of R16(*A*), R38(*B*) and R194(*C*) of wild-type 7α-HSDH from *Clostridium absonum*.

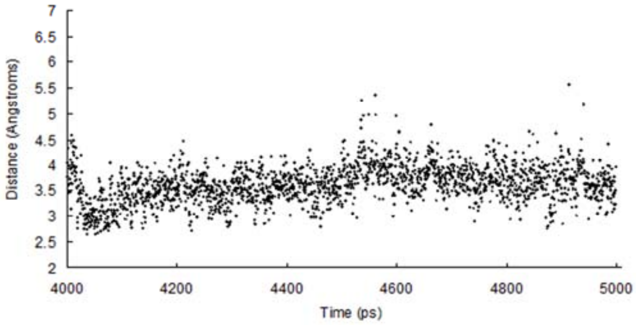

Fig. S11. Donor-acceptor distances of hydrogen bond between the guanidinium of Arg38 and 2′-phosphate of adenine ribose of $NADP^+$ in the mutant R16G of 7α-HSDH from *Clostridium absonum*.

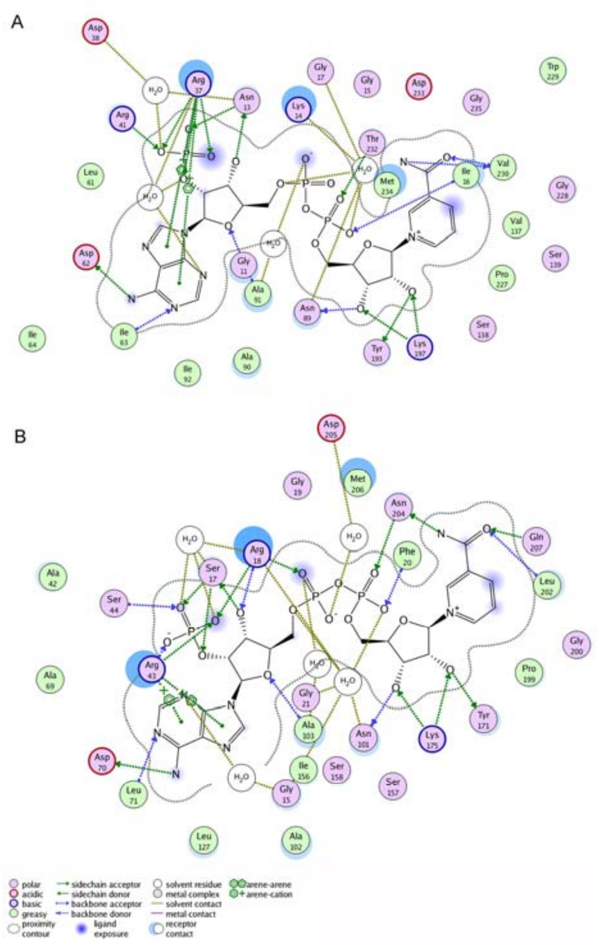

Fig. S12. Interactions between NADP(H) and enzymes. (*A*) *Sus scrofa* carbonyl reductase/20beta-hydroxysteroid dehydrogenase (PDB code: 1N5D). (*B*) *Mus musculus* Sepiapterin reductase (PDB code: 1NAS).

Table S1 Statistics of data collection and processing

Data collection	*CA* 7α-HSDH/$NADP^+$/TCDCA
Diffraction source	RIGAKU FR-E^+
Wavelength (Å)	1.54
Temperature (K)	100
Detector	R-AXIS IV++
Crystal-detector distance (mm)	160
Rotation range per image (°)	0.5
Total rotation range (°)	180
Exposure time per image (s)	200
Space group	$P2_12_12_1$
Unit cell parameters	
a, *b*, *c*(Å)	67.18, 69.68, 220.39
α, β, γ (°)	90.0, 90.0, 90.0
Total No. of reflections	469741
No. of unique reflections	69768
Completeness (%)	98.3
Redundancy	6.7
$I/\sigma(I)$	7.1

Table S2 Statistics of structure solution and refinement

Data refinement	*CA* 7α-HSDH/NADP$^+$/TCDCA
Resolution range (Å)	50-2.0
R_{factor} (%)	18.2
R_{free} (%)	22.2
Rms deviation from ideal geometry	
Bonds (Å)	0.0187
Angles (°)	1.5646
Ligands	
NADP$^+$	4
TCDCA	4
Glycerol	4
Average *B* factor (Å^2)	
Protein	14.13
NADP$^+$	38.11
TCDCA	27.18
Glycerol	17.10
Water	26.71
Ramachandran plot	
Most favoured (%)	97.7%
Allowed (%)	1.6%

Table S3 Residues interacting with the 2'-phosphates of adenine ribose of NADP(H) in 11 SDRs

PDB code	Enzyme	Residues
5EPO	7α-HSDH (*C. absonum*)	Thr15, Arg16, Arg38, Arg39
1CYD	Mouse lung carbonyl reductase	Lys17, Arg39, Thr40
1QYV	Human estrogenic 17β-HSDH	Ser11, Arg37
1DOH	Trihyroxynaphthalene reducatase (*M. grisea*)	Arg39, Ala61, Asn62, Ser63
1N5D	Porcine testicular carbonyl reductase	Asn13, Arg37, Arg41
1EDO	β-keto acyl carrier protein reductase (*B. napus*)	Ser26, Arg27, Ala49, Arg50, Ser51
1OAA	Mouse sepiapterin reductase	Ser17, Arg18, Arg43, Ser44
2AE2	Tropinone reductase-II (*D. stramonium*)	Arg19, Arg41
1H5Q	Mannitol dehydrogenase (*A. bisporus*)	Asn20, Arg21, Arg43, Ser44, Ala45
1YBV	1,3,8-Trihydroxynaphthalene reductase (*M. grisea*)	Arg39, Ala61, Asn62, Ser63
1Y5M	Murine 11beta-HSDH	Ser43, Arg66, Ser67

Table S4 The free binding energies (kcal/mol) between enzymes (WT and R16G) and ligands (TCDCA and $NADP^+$) in the two ternary complexes

Complex	Ligand	ΔG binding
WT-TCDCA-$NADP^+$	TCDCA	-45.40 (4.38)
	$NADP^+$	-99.84 (7.13)
R16G-TCDCA-$NADP^+$	TCDCA	-41.19 (3.80)
	$NADP^+$	-72.99 (7.78)
R38D-TCDCA-$NADP^+$	TCDCA	-34.44 (4.36)
	$NADP^+$	-76.83 (7.54)

The values of standard deviations (SD) are in parentheses.

Table S5 Primers used for site-directed mutagenesis using overlapping PCR

Mutation	Template for PCR	Mutagenic forward primer	Mutagenic reverse primer
A37D	codon-optimized 7α-HSDH	5'-CTGGTGTATCTGGCAGATCGTAGCGAGGAACTG-3'	5'-CAGTTCCTCGCTACGATCTGCCAGATACACCAG-3'
A37D/R38I	A37D	5'-CTGGTGTATCTGGCAGATATTAGCGAGGAACTG-3'	5'-AGCCAGTTCCTCGCTAATATCTGCCAGATACAC-3'
A37D/R38Y	A37D	5'-CTGGTGTATCTGGCAGATTATAGCGAGGAACTG-3'	5'-AGCCAGTTCCTCGCTATAATCTGCCAGATACAC-3'
A37D/R38V	A37D	5'-CTGGTGTATCTGGCAGATGTTAGCGAGGAACTG-3'	5'-AGCCAGTTCCTCGCTAACATCTGCCAGATACAC-3'
